# Altering Microbiomes with Hydroxyapatite Nanoparticles: A Metagenomic Analysis

**DOI:** 10.3390/ma15175824

**Published:** 2022-08-24

**Authors:** Vuk Uskoković, Victoria M. Wu

**Affiliations:** 1 TardigradeNano LLC, Irvine, CA 92604, USA; victoriamwu1015@gmail.com; 2 Department of Mechanical Engineering, San Diego State University, San Diego, CA 92182, USA

**Keywords:** bacterial microbiome, metagenomics, nanoparticles, 16S ribosomal next-generation sequencing, environmental remediation

## Abstract

Hydroxyapatite (HAp), the most abundant biological material among mammals, has been recently demonstrated to possess moderate antibacterial properties. Metagenomics provides a series of tools for analyzing the simultaneous interaction of materials with larger communities of microbes, which may aid in optimizing the antibacterial activity of a material such as HAp. Here, a microbiome intrinsic to the sample of sandy soil collected from the base of an African Natal plum (*Carissa macrocarpa*) shrub surrounding the children’s sandbox at the Arrowhead Park in Irvine, California was challenged with HAp nanoparticles and analyzed with next-generation sequencing for hypervariable 16S ribosomal DNA base pair homologies. HAp nanoparticles overwhelmingly reduced the presence of Gram-negative phyla, classes, orders, families, genera and species, and consequently elevated the relative presence of their Gram-positive counterparts. Thermodynamic, electrostatic and chemical bonding arguments were combined in a model proposed to explain this selective affinity. The ability of amphiphilic surface protrusions of lipoteichoic acid in Gram-positive bacteria and mycolic acid in mycobacteria to increase the dispersibility of the bacterial cells and assist in their resistance to capture by the solid phase is highlighted. Within the Gram-negative group, the variability of the distal, O-antigen portion of the membrane lipopolysaccharide was shown to be excessive and the variability of its proximal, lipid A portion insufficient to explain the selectivity based on chemical sequence arguments. Instead, flagella-driven motility proves to be a factor favoring the evasion of binding to HAp. HAp displayed a preference toward binding to less pathogenic bacteria than those causative of disease in humans, while taxa having a positive agricultural effect were largely captured by HAp, indicating an evolutionary advantage this may have given it as a biological material. The capacity to selectively sequester Gram-negative microorganisms and correspondingly alter the composition of the microbiome may open up a new avenue in environmental and biomedical applications of HAp.

## 1. Introduction

The bacterial microbiome presents the foundation of all higher forms of life. Its sentient supremacy on planet Earth notwithstanding, *Homo sapiens* is, compositionally, more microbial than it is human, given that 90% of cells in a human body are bacterial and the number of genes in it are over 99% bacterial [1,2]. The complexities intrinsic to this biological domain are best illustrated by the fact that it took 3 billion years for the first bacterium to evolve into a protozoan, 250 million years for protozoa to evolve into animals and the same duration of time for these first animals to evolve into mammals [3,4]. Here, the microbiome is not a simple stochastic congregation of various bacterial species but rather a community of microbes built and sustained on intricate biophysical and biochemical communication networks.

Whereas the health of the soil has been associated with the health of its microbiome since the earliest days of agricultural science, only in the last decade have we started to witness significant strides in the understanding of bacterial infection as the disease of the microbiome. This new paradigm appears to be slowly prevailing over the classical model summed in Louis Pasteur’s and Robert Koch’s ‘one bug, one disease’ tenet [5], tying the origin of infectious disease to a comparatively simplistic proliferation of a single bacterial species against the host organism’s immune protection. Repercussions of this paradigm shift are far-reaching, but common to all of them is pointing implicitly at the symbiosis between the eubacterial and the animal kingdoms, the deterioration of which leads to the onset of infection [6,7] or of a seemingly unrelated medical condition [8,9,10]. With the shift to the microbiome model of infectious disease, it has become clear that conditions favoring the suppression or proliferation of single or multiple of metabolically interconnected species constituting the healthy biome can alter the biotic balance and induce dysbiosis typified by the unhampered spread of a single dominant species with pathogenic features [11,12,13]. Conversely, infection with a single pathogenic bacterium can occasionally restore the normal microbiome independently of the antibiotic treatment [14]. The prediction of these events, however, requires the employment of sophisticated statistical frameworks of analysis, necessitating a shift in the technical language to accompany this paradigm shift. Still, although it is now known that microbiome characteristics are determinants of whether one pathogenic population would take over the host organism, we are still far from using the microbiome therapeutically, such as by having patients undergo injection of cocktails of bacteria at the site of their chronic infection, be it as a treatment or a prophylaxis, in just about the same way as probiotics are used to improve the health of the digestive tract. To that end, promising studies involving the attenuated strain inoculation of infants infected with highly virulent bacterial strains dating back to the 1960s have recently been revived in light of the rising interest in the concept of microbiome manipulation to treat the existing bacterial infections or prevent the future ones [15]. Before this therapeutic idea becomes a reality, however, it is essential to understand how specific biomedical materials interact with the microbiomes of different origins.

Hydroxyapatite (HAp, Ca_5_(PO_4_)_3_OH) is the most abundant inorganic material in the mammalian bodies, where it forms hard tissues, including bone, enamel, dentin and cementum, but also other organs, including tusks in animals such as elephants or narwhals, horns in deer, moose, elks and caribous, and the shell-like osteoderm of armadillos [16]. Despite being one of the oldest biomaterials in research and clinical application, HAp continues to attract the attention of researchers to this day, and new studies on it do not abate [17,18,19]. Recently, our group demonstrated that HAp displays finite antibacterial properties, which appear to become intensified when the bacterial species are converted from regular lab strains to clinical, multidrug-resistant ones [20,21,22]. Interestingly, when it comes to mammalian organisms, HAp, such as that comprising the dental tissues, is in contact with a rich microbiome of the oral cavity, while many infective pathologies of the maxillofacial area, such as periodontitis [23] or dental caries [24], are increasingly being seen as diseases of the microbiome. However, it is virtually unknown how HAp affects the microbiomes that it comes in contact with. To this date, it has not been studied which exact populations in any given microbiome are being reinforced by HAp and which are being suppressed.

In this study, we take to the task of analyzing the interaction of HAp with the microbiome present in a sample of sandy soil. Studying the interaction of HAp with the soil microbiome is justified firstly by the fact that HAp has a long history of use in soil [25] and wastewater [26] remediation, which owes to the biocompatibility and environmental friendliness of this material [27], but also to its capacity to act as a universal sorbent [28]. The typically high specific surface area of HAp and its ability to undergo ion exchange with metal ion contaminants in water or soil have rendered this material a highly reactive sorbent [29]. In addition to its affinity for ionic species, HAp also effectively adsorbs organic molecules, for which reason it is still being used as a major chromatographic support and a purification medium in preparative biochemistry and other branches of the biotech industry [30], well over half a century since it was proposed for this role [31]. Moreover, HAp is a type of material susceptible to alteration of its surface composition and structure, be it via chemical [32] or physical [33,34] effects, which, in turn, affects the biological response, rendering the material tailorable for exhibiting a broad spectrum of effects on biological systems of various origins. Here, the soil microbiome can be perceived as a solid model system for analyzing the general trends in the interaction of HAp with a community of interrelated microorganisms present in virtually every realistic microbe-infested biological system.

To study the interaction with this model microbiome, next-generation sequencing (NGS) of the genome was implemented. NGS presents a state-of-the-art tool in metagenomics, enabling the study of diverse microbiome communities in their native milieus of various origins, including the clinical [35], the agricultural [36], the geochemical [37], and the extraterrestrial [38]. By offering the insight into the global gene expression of a community of microorganisms, NGS circumvents the need for culturing the often uncultivable microorganisms and allows for the discernment of critical population dynamics within this community. This information can be crucial for determining the relationship between the resident microbiome and invasive pathogens, with direct repercussions on the host–pathogen interaction [39]. The findings of this study will provide an answer to the question if and, if so, how HAp affects a model microbiome. Whether the amplicons of one taxa will be suppressed over those of others and whether a definite trend will be deducible from these effects, such as that predisposing microorganisms with certain phenotypic features to be affected more than those with others, are some of the questions that this study has attempted to answer. Moreover, the approach used in the analysis of the raw NGS data has not emulated the methodology of any prior metagenomic analysis. As such, it can be an instructive model for numerous future studies on similar systems of interest to follow.

## 2. Materials and Methods

The synthesis of HAp (Ca_5_(PO_4_)_3_OH) nanoparticles proceeded by adding 100 mL of 0.1 M aqueous solution of calcium nitrate (Ca(NO_3_)_2_, Fisher Scientific, Waltham, MA, USA) containing 12.5 mL 28% NH_4_OH dropwise to the same volume of 0.06 M aqueous solution of monoammonium phosphate (NH_4_H_2_PO_4_, Fisher Scientific) heated to 80 °C and containing 6.25 mL 28 vol.% ammonia (NH_4_OH, Sigma Aldrich, Burlington, MA, USA). After the addition of Ca(NO_3_)_2_ to the phosphate solution vigorously stirred with a magnetic bar (400 rpm) had been completed, stirring was suspended, and the precipitate was left to age in atmospheric conditions together with its parent solution for 3 h. After the given time, the precipitate was centrifuged (5 min at 3500 rpm) and the supernatant was decanted. The precipitate was then washed with deionized (DI) H_2_O, separated from the supernatant by centrifugation, and dried overnight under the ambient conditions. A detailed characterization of the resulting powder, which consisted of rod-shaped nanoparticles with the widths in the 5–10 nm range and the lengths of up to 100 nm, is reported elsewhere [40,41,42].

Antibacterial assays reported here were performed against two laboratory strains of Gram-negative bacteria, namely *Pseudomonas aeruginosa* (ATCC27853, Carolina Biological, Burlington, NC, USA) and *Escherichia coli* (ATCC14948, Carolina Biological), and three laboratory strains of Gram-positive bacteria, namely *Staphylococcus aureus* (ATCC27661, Carolina Biological), *Staphylococcus epidermis* (ATCC12228, Carolina Biological), and *Enterococcus faecalis* (ATCC29212, Carolina Biological). These assays were also performed against one multidrug-resistant (MDR) strain of a Gram-negative bacterium, namely *Pseudomonas aeruginosa* (gift of J. Yamaki), and one MDR strain of a Gram-positive bacterium, namely methicillin-resistant *S. aureus* (MRSA, gift of J. Yamaki). Bacteria were grown separately overnight in either Luria broth or Vegatone broth at 37 °C. HAp was added as a powder at different concentrations to 1 mL of 1:50 diluted overnight culture of bacteria, and the suspension was incubated overnight at 37 °C. After the overnight incubation, 200 μL of the bacterial broth was centrifuged and resuspended in the same volume of an aqueous solution of 110 mM NaCl and 250 mM HCl to dissolve HAp and avoid its interference with the optical density (OD) measurements. The OD measurements were performed at the wavelength of 600 nm on a BMG LABTECH FLUOstar Omega spectrophotometer. IC_50_ values were deduced from the exponential decay fits of the OD vs. HAp concentration plots in the 0–100 mg/mL range.

The sandy soil sample was collected from the base of the southernmost of the six African Natal plum (*Carissa macrocarpa*) shrubs surrounding the children’s sandbox at the Arrowhead Park in the Woodbridge area of Irvine, California. Another control sample to be used specifically in the data variance analysis was collected from a swamp in Encino, 60 miles northwest of Irvine. The sandy soil from the Arrowhead Park was treated in an orbital shaker and deionized water at the concentration of 100 mg/mL with 5 mg/mL HAp for 24 h. After the given period of time, the solid phase was spun down by centrifugation. Cell lysis and bacterial DNA were isolated, and the 16S metagenomic library was created using the Ion 16S^TM^ Metagenomics Kit (A26216, Thermo Fisher Scientific, Waltham, MA, USA), according to the manufacturer’s instructions. The NGS analysis was performed on an Ion Torrent sequencer (Thermo Fisher Scientific) using standard sequencing protocols. Raw NGS data were analyzed for principal components and population profiles with the use of the Ion Reporter software (Thermo Fisher Scientific) in its default settings. The software used preconfigured and manually customizable workflows optimized for Ion AmpliSeq panels to identify genomic variants that included single-nucleotide polymorphisms, copy number variations, indels, and gene fusions. Ion Reporter used annotations from dozens of publicly available databases, including COSMIC, dbSNP and OMIM, and it had the inbuilt code that assisted in the automated filtering of the most relevant genetic variants. Standard analytical settings and the good high-quality read count relative to the total ensured that taxonomic population quantities were statistically comparable, regardless of the data presentation based on significant digit rounding and absence of error bars, which is customary in NGS literature [43,44,45].

## 3. Results and Discussion

### 3.1. Study Design

In the reported metagenomic analysis, a sandy soil sample collected from the base of an African Natal plum shrub adjacent to a children’s playground sandbox was compared against the same sandy soil sample treated with HAp nanoparticles. To measure the global genetic expressions within the microbiome, the following primers to hypervariable regions of the 16S ribosomal 1542 base pair gene encoding for small subunit ribosomal RNA were used: primer set V2-4-8 and primer set V3-6,7-9. This gene is highly conserved, but its variable regions (V1–V9) vary across the Bacteria domain. Whereas 18S ribosomal DNA amplicon sequencing is used for eukaryotic organisms, the 16S analogue is the standard for the prokaryotic ones [46]. A typical workflow for the NGS of the 16S ribosomal gene was followed here, involving first the DNA extraction and confirmation of its purity and concentration, and then the PCR amplification of the variable regions of the 16S gene and creation of the 16S metagenomic library, after which came sequencing, and, finally, the identification of detected sequences based on similarity matching with respect to the reference 16S gene sequences. Although this method is highly discriminatory, considerable homologies, >99%, still exist for a number of bacterial genera.

Usually, incubation times of one month or longer are used in toxicological studies of soils exposed to nanoparticles [47,48,49], but since toxicological effects are not the focus of this study, the incubation time was significantly shorter. Because 24 h is the typical time window for the action of antibacterial agents in biological organisms before they become metabolized or excreted [50], the emphasis in the experimental design was on the ability of HAp to produce effects on the microbiome that take place within this period of time. Detecting such short-term genomic effects is more challenging than detecting effects taking place over longer time scales. Moreover, in genomic sequence analyses in general and especially at this short of a treatment time scale, the antibacterial effect of nanoparticles or any other agents per se could not be determined unambiguously. This is because even a lethal effect of the agent on a particular bacterial population would not remove the genetic footprint of the given bacteria from the system. One way around this issue is to couple a live/dead assay such as the propidium monoazide one to genomic sequencing to yield the difference between the live and the total microbiota [51]. Lest the expediency and practicality of the method be compromised, such additional assays were not used in this study.

In short, the bacterial inhibition effects observed here are bound to be negligible compared to those occurring over longer time scales. However, HAp is known to be an excellent sorbent of biological molecules and microorganisms, including the bacterial cells, and this sorption reaches saturation well within the 24 h time window. This removal of specific bacterial taxa from the system via physisorption is, therefore, expected to present the major mechanism behind the dampening of specific amplicons by the treatment with the nanoparticles.

### 3.2. Analysis of the Global Effects

**Figure 1:**
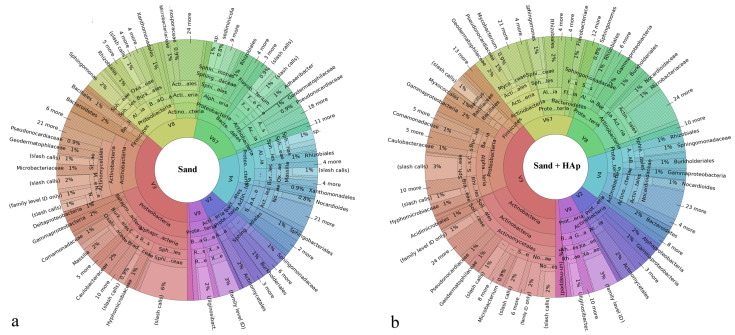
Krona plots representing the microbiome composition of the sandy soil before (**a**) and after (**b**) the treatment with HAp nanoparticles sorted out by primers (V2-4-8 and V3-6,7-9) targeting different variable regions of the 16S ribosomal gene.

**Figure 2:**
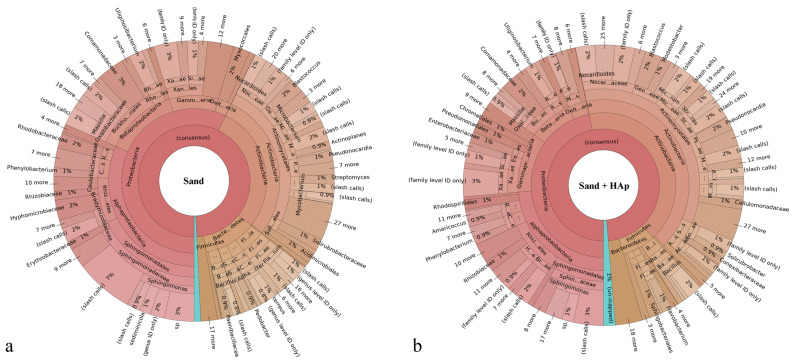
Consensus Krona plots representing the microbiome composition of the sandy soil before (**a**) and after (**b**) the treatment with HAp nanoparticles.

Global, high-target resolution Krona plots sorted out by primers for the untreated soil and the soil treated with HAp nanoparticles are shown in Figure 1. Fusion primers V2-4-8 and V3-6,7-9 targeting the hypervariable 16S ribosomal DNA regions were used, and a large number of targets were detected in each primer region. Only 1 to 2% of the sequences were unmapped, suggesting both a highly selective and highly sensitive analytical process. Slash calls are different from indiscriminate calls because of their representing two or more possible taxa, but they still do present ambiguous reference targets. Such ambiguous calls were present solely at the lower taxonomic levels of genera and species. Out of all the variable regions of the 16S gene, those targeted by the V3 primer gave the largest percentage of correctly allocated genera and species. Krona plots sorted out for the primer output are more informative for the purpose of confirming the specificity and sensitivity of the analysis, while the corresponding plots representing the consensus information across all the primers combined present more meaningful plots for the comparison of the collective gene expression. Such consensus plots for the untreated soil and the soil treated with HAp nanoparticles are shown in Figure 2.

**Figure 3:**
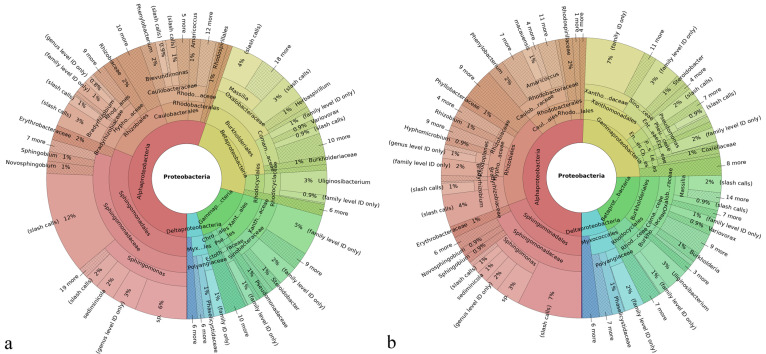
Krona plots focused on the most abundant of all phyla in the untreated (**a**) and treated (**b**) soil samples, namely Proteobacteria.

Microorganisms forming the microbiome community are classified within a taxonomy comprising eight hierarchical levels, which are as follows, from the lowest to the highest: species, genus, family, order, class, phylum, and domain and/or kingdom. Correspondingly, the Krona plots assign operational taxonomic units (OTUs) detected in the system to different levels of this hierarchy, where the closer one is to the center, the higher the hierarchical level is. For example, as seen in Figure 2a, the most abundant and highest taxonomic unit present in the soil was that of Proteobacteria, which is a phylum within the Bacteria domain representing Gram-negative bacteria only. The most abundant class within this phylum was that of Alphaproteobacteria and the most abundant order within this class was that of Sphingomonadales. Further down the hierarchy, the most abundant family within this order was that of Sphingomonadaceae, while *Sphingomonas* was its most prominent genus in the soil. Finally, within this genus, the most prominent species was *Sphingomonas sediminicola*, which represented 16% of population within the *Sphingomonas* genus and 1% of the total species making up the soil. Characterized by low virulence, only one of the *Sphingomonas* species is known to be a commensal, opportunistic pathogen, namely *Sphingomonas paucimobilis*, which has been reported to elicit infection in immunocompromised individuals only [52]. Figure 3 shows the magnified regions of specifically this Proteobacteria phylum for the treated and the untreated soils.

**Figure 4:**
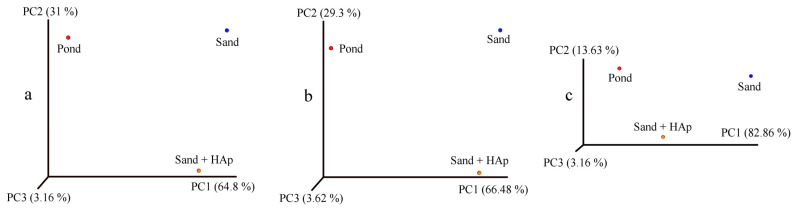
Scatter plots showing the results of the Euclidean principal coordinate analysis (PCA) indicating variance of the microbiome composition at the level of families (**a**), genera (**b**) and species (**c**), before and after the treatment with HAp nanoparticles in comparison with the composition of the soil sampled from a random location, a pond in the Encino area of Los Angeles. Principal components PC1, PC2 and PC3 ranged from 64.8–82.9%, 13.6–31.0%, and 3.2–3.6%, respectively.

Before detailed effects of the treatment at each individual taxonomic level are discussed, a few global effects need to be mentioned. First, principal coordinate analysis (PCA) plots are shown in Figure 4, each representing a set of uncorrelated orthogonal axes measuring the variability of the comparative datasets with respect to three lowest taxonomical levels, namely family, genus and species. To more precisely assess the variability of the soil microbiome before and after the treatment, an additional control in the form of a soil sampled out from a different location, a pond in the Encino area of Los Angeles, was added to the analysis. Given that objects ordinated closer to each other are more similar than those ordinated farther away [53], the approximate equidistance between the positioning of the control soil sample (i.e., pond), the untreated soil sample (i.e., sand) and the treated soil sample (i.e., sand + HAp) suggests that the variability between the microbiome composition of all three specimens is considerable and similar in magnitude. In other words, the treatment with HAp nanoparticles makes the sandy soil more or less as different from the untreated sandy soil sample as this untreated sandy soil is different from a control swampy soil sampled from a wholly different location. These results demonstrate that by uptaking and removing specific bacteria from the soil, HAp nanoparticles considerably alter the composition of the microbiome. The changes produced thereby are as prominent as those immanent in the natural variability of the microbiome composition in soils from two totally different geographical areas.

**Figure 5:**
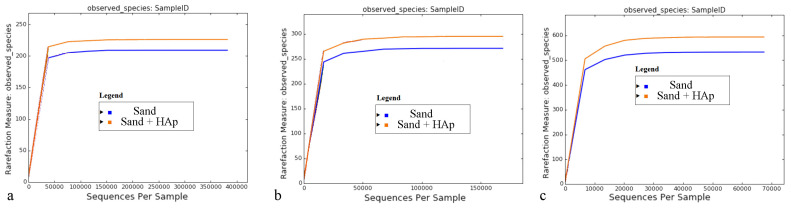
Rarefaction curves showing the total numbers of families (**a**), genera (**b**) and species (**c**) detected in the soil sample before (blue curves) and after (orange curves) the treatment with HAp nanoparticles.

Another global effect caused by the addition of HAp nanoparticles to the soil is that of a mild increase in the total number of OTUs corresponding to bacterial families (Figure 5a), genera (Figure 5b) and species (Figure 5c) in the microbiome, which was equivalent to 7.0%, 8.0% and 13.4%, respectively. HAp per se, of course, could not increase the diversity of the soil in the absence of the openness of the system to other bacterial sources. The effects of the release of calcium and phosphate ions or the elevation of the alkalinity of the soil through the release of hydroxyl ions could alter the composition of a chemically, if not thermodynamically, closed microbiome system, but such effects could not lead to the enhanced diversity of the microbiome. HAp was synthesized in the chemical safety cabinet using glove protection and autoclaved glassware, but even under these conditions, the solution and the obtained powder become exposed to air particulates during the separation from the solution and the drying, adding to the use of nonsterile reactants, which can be a logical candidate for the reason behind this enhancement of the bacterial diversity of the microbiome. However, since the amplified populations due to the presence of HAp, as will be revealed shortly in the text, are soil-specific, this has suggested that the increase in the total number of OTUs detected after the treatment with HAp is not due to contamination but rather due to the amplification of less abundant taxa, which are otherwise being masked by their more prominent counterparts. The removal of the latter microorganisms by their selective binding onto HAp is the likely mechanism leading to a more copious detection of the less abundant species comprising the microbiome and their increased presence when the latter is expressed in relative units, specifically percentages. Another global effect deducible from the reaching of the saturation plateau of all rarefaction curves shown in Figure 5 is that virtually all taxonomic units detectable in the microbiome were distinguished.

### 3.3. Results on the Most Abundant Taxa

**Figure 6:**
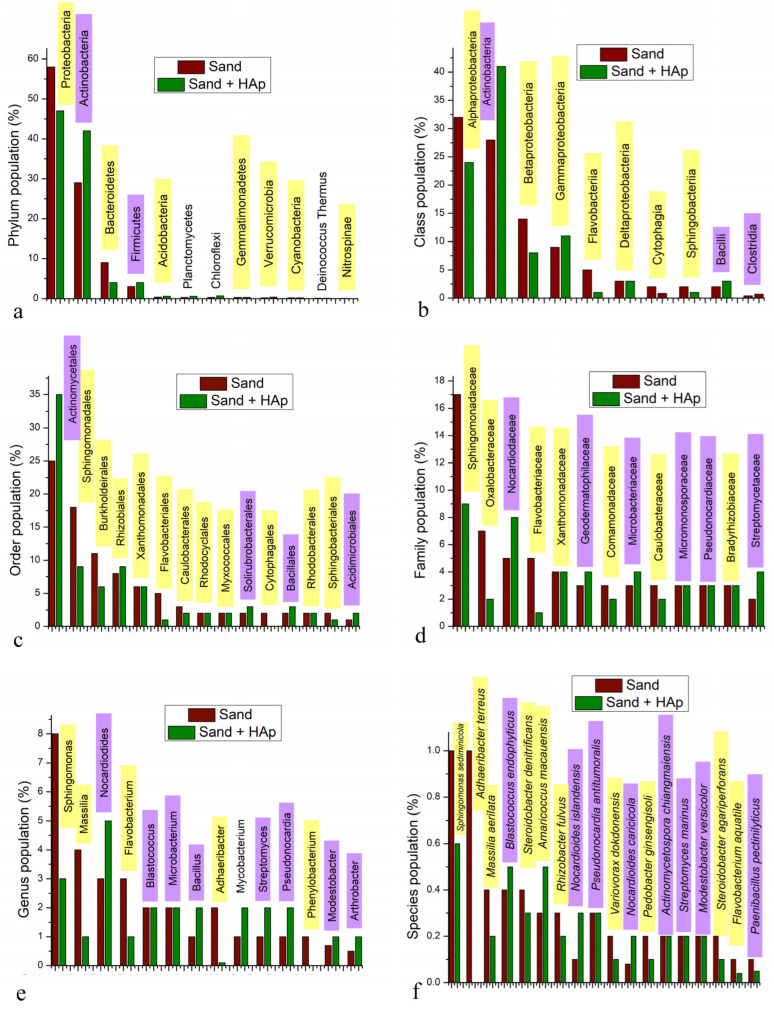
Relative presence of the most dominant phyla (≥0.05%) (**a**), classes (≥0.5%) (**b**), orders (≥2%) (**c**), families (≥3%) (**d**), genera (≥1%) (**e**) and species (≥0.1%) (**f**) in the sandy soil microbiome before and after its treatment with HAp nanoparticles. Gram-negative taxa are framed in yellow, while Gram-positive taxa are framed in violet.

Dividing the total makeup of the microbiome to the most abundant individual phyla, classes, orders, families, genera and species shows that some of them increase and some decrease in terms of percentages following the incubation with HAp nanoparticles (Figure 6). A comparison at the level of phyla shows that the most abundant phylum, namely, Proteobacteria, becomes distinctly reduced in population upon the incubation with HAp, from 58 to 47% of the total makeup of the microbiome (Figure 6a). The same population reduction effect was seen for the third most abundant phylum, namely Bacteroidetes, which dropped by more than twofold, from 9 to 4% upon the incubation with HAp. Clearly, this population reduction effect is due to the microbial adsorption onto HAp and the effective removal of the given microorganisms from the system with centrifugation, but also, potentially, albeit to a far lesser extent, because their lysis may be less complete when the cells are protected by the nanoparticles that they adsorb onto. Interestingly, both of these phyla displaying the affinity for HAp are Gram-negative. In contrast, the second most prominent phylum, namely Actinobacteria, which is a Gram-positive phylum, increased in relative presence after the incubation with HAp, from 29 to 42% of the total microbiome composition. The fourth most prevalent phylum, Firmicutes, is mostly Gram-positive, and its relative population also increased after the incubation with HAp, reiterating the trend of the selective affinity of HAp for Gram-negative bacteria. Among the phyla detected in amounts lower than 1% in the soil microbiome, however, this trend was not present. In fact, the relative presence of all but two of these minor phyla in the microbiome increased through the treatment with HAp. These two exceptions were Crenarchaeota (the least abundant phylum at 0.002%) and Nitrospirae (0.03%). Agreeing with the overall trend of the selective removal of Gram-negative species, these two phyla fully removed from the system are Gram-negative. In addition, due to the removal of more prominent phyla and the resulting exposure of the less prominent ones, a few phyla undetected in the untreated soil became detectable after the treatment: Spirochaetes (0.03%), Amatimonadetes (0.02%), Thermodesulfobacteria (0.02%), and Aquificae (0.01%).

By selectively binding onto Gram-negative bacteria from the Proteobacteria and Bacteroidetes phyla, the prominence of the second and the fourth most abundant phyla in the soil microbiome, which happened to be Gram-positive Actinobacteria and Firmicutes, was magnified. Since Actinobacteria are mostly soil-dwelling microorganisms [54], this has eliminated the possibility that the detected increase in the total number of phyla—and, further down the taxonomy, species—could be due to contamination, conforming to the previous argumentation. Rather, by removing selected phyla from the system, the relative presence of the microbes left intact naturally increased. This means that even the evident increase in the relative population of phyla present at under 1% of the total microbiome could be a simple artifact of the removal of more prominent phyla from the system by HAp, primarily Proteobacteria. Overall, these results are also in agreement with the earlier detected greater antibacterial activity of HAp against Gram-negative bacteria as compared to the activity with respect to Gram-positive ones [20,21,22]. These results are summarized in Table 1, demonstrating the ability of HAp to not only remove but also inhibit planktonic Gram-negative microorganisms from aqueous systems. Clearly, the adsorption of the given microbes evidenced here is a prerequisite for the accomplished inhibition of their growth.

With regard to the comparison of bacterial classes, no new class appeared due to the presence of HAp. As consistent with the results obtained from the analysis of effects on phyla, HAp decreased the prevalence of all of the most prevalent classes (≥0.5%) of Gram-negative bacteria except for Gammaproteobacteria, whose level increased with the addition of HAp, and Deltaproteobacteria, whose level remained unchanged (Figure 6b). In fact, as can be seen from Figure 3, the treatment with HAp led to the replacement of Betaproteobacteria as the second most prevalent class in the Proteobacteria phylum with Gammaproteobacteria. Other classes belonging to the Proteobacteria phylum, including Alphaproteobacteria and Betaproteobacteria, all decreased in prominence after the treatment with HAp. Gammaproteobacteria are a class that positively correlates with soil pollution and with the abundance of antibiotic-resistant genes in the microbial population [55]. It is also a category to which a greater number of pathogenic species causative of disease in humans belong as compared to other Proteobacteria classes, including the likes of *Pseudomonas aureginosa*, *Escherichia coli*, *Francisella tularensis*, *Yersinia pestis*, *Salmonella enterica*, *Shigella dysenteriae*, *Legionella pneumophila*, *Coxiella burnetii*, and *Vibrio cholerae*. Because of the taxonomic congruence between the phylum and the class of Actinobacteria, the effect of HAp was that of an increase in the population of them both, which was consequential to the affinity of HAp for Gram-negative Proteobacteria. The other two Gram-positive classes of bacteria counting among the most prominent ones in the microbiome, namely Bacilli and Clostridia, also increased in prominence with the presence of HAp, from 2 to 3% and from 0.4 to 0.7% of the total microbiome, respectively. As far as other Gram-negative classes are concerned, Flavobacteriia was removed more intensely than any other class, fivefold, from 5 to 1% of the total microbiome, as well as Cytophagia, which was reduced from 2 to 0.8%.

Considering the trend applying to bacterial orders, it complied with that detected for phyla and classes. Namely, the population prevalence of all four Gram-positive orders present at 2% or more relative to the total composition of the microbiome increased after the treatment with HAp (Figure 6c). In contrast, the populations of six out of 11 Gram-negative orders present at 2% or more relative to the total composition of the microbiome decreased after the treatment with HAp; four out 11 of these orders remained at the same level; and the concentration of only one out of 11 orders increased with the treatment. The most intense population reduction was seen for the following: Flavobacteriales, which dropped fivefold, from 5 to 1% per the total microbiome composition; the most prevalent order within the most prevalent phylum, namely Sphingomonadales, which dropped twofold, from 18 to 9%; and Burkholdeirales, which dropped almost twofold, from 11 to 6%. Cytophagales, the most prominent member of the Cytophagia class, was the only order present at 2% or more that was completely eliminated from the system after the treatment with HAp.

Among the most prominent bacterial families in the soil, the same trend showing the reduction in the population of Gram-negative microorganisms from the system by the treatment with HAp was being overwhelmingly present (Figure 6d). At higher taxa, there were exceptions to this trend, albeit rare, but at the family level, no exceptions were detected. Specifically, among 13 families present at concentrations equal to or higher than 3% in at least one of the sample groups (treated vs. untreated), five out of seven Gram-negative families decreased in relative population with the treatment and two remained at the same level, while four out of six Gram-positive families increased in relative population with the treatment and two remained at the same level. At the genus level, an even higher level of selectivity was observed among populations being equal to or exceeding 1% per the total microbiome composition, with the relative population of all five of the Gram-negative genera decreasing with the treatment and the relative population of six out of eight Gram-positive genera increasing and two out of these eight remaining constant (Figure 6e).

**Figure 7:**
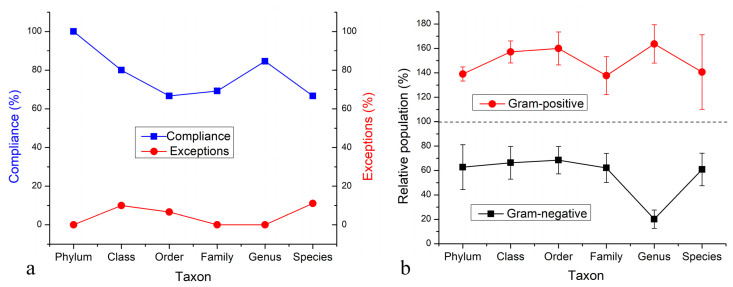
Percentage of taxa conforming to the trend of HAp decreasing the relative presence of Gram-negative populations and increasing the relative presence of Gram-positive populations (blue line), and the percentage of exceptions to this trend (red line) (**a**). The two values expressed in percentages must have the percentage of calls that were equal for the untreated and treated sample groups added to them to reach 100%. Average change in the relative population of a Gram-negative and a Gram-positive taxon for each hierarchical level of the classical nomenclature relative to the untreated control (**b**). Data points in (**b**) represent averages, while error bars represent the standard errors of the mean. Dashed line in (**b**) represents the 100% concentration of the control. All phyla, classes, orders, families, genera and species whose concentrations in the microbiome were higher than or equal to 1, 0.5, 2, 3, 1, and 0.1%, respectively, in either the treated or untreated soils are included among the data points.

Data obtained at the most fundamental level, that of bacterial species, need to be taken with the most reservation because a number of bacteria were detected as unspecific “sp.”, adding to the slash calls and genus-only calls to produce a greater level of indiscriminateness than that present at higher taxa. Therefore, Figure 6f lists only the most prominent species detected unambiguously in the microbiome, with a full species name, which, based on these arguments, need not be taken as the most prominent ones in the real system. Still, they are illustrative of the overall trend produced by HAp nanoparticles in interaction with the soil microbiome. Namely, as shown in Figure 7a, there is an overwhelming tendency for HAp to remove Gram-negative bacteria from the microbiome, thus boosting the relative presence of their Gram-positive counterparts. This tendency was present at all taxonomic levels, but it was most pronounced at the level of phyla, where 100% of phyla present at 1% or more complied with the trend and no exceptions were noted, and at the level of genera, where 84.6% of genera conformed to the trend and no exceptions were noted. At no taxonomic level did the percentage of exceptions, in fact, exceed the maximal value of 11.1%, which applied at the species level. Correspondingly, as seen in Figure 7b, the average reduction in the relative presence of a prominent Gram-negative taxon in the soil due to the treatment with HAp was down to between 20.2 and 68.5% of the control, whereas the average enhancement in the relative presence of a prominent Gram-positive taxon due to the same treatment ranged between 137.8 and 163.7% of the control. At the lowest level of the classical nomenclature, that of species, the presence of Gram-negative species was reduced by more than one-third, i.e., down to 60.8%, while that of Gram-positive species increased by more than one-third, i.e., up to 140.6%. The coinciding of the maximum of the Gram-positive curve and the minimum of the Gram-negative curve in Figure 7b is yet another indicator that the enhancement of the relative presence of Gram-negative species is a direct consequence of the selective sequestration of Gram-negative bacteria by HAp nanoparticles. It is uncertain, however, why the overlap of the given maxima and minima occurs at the genus level, unless the most optimal combination of diversity and non-homology at this level presents the decisive factor. This may be explained by the fact that the taxonomical levels higher than the genus exhibit a lower diversity of potential calls, while the only taxonomic level higher than that of genera, namely the species, exhibits the highest density of indiscriminate calls due to sequence homologies.

### 3.4. Analysis of the Effect on Potential Human Pathogens

**Figure 8:**
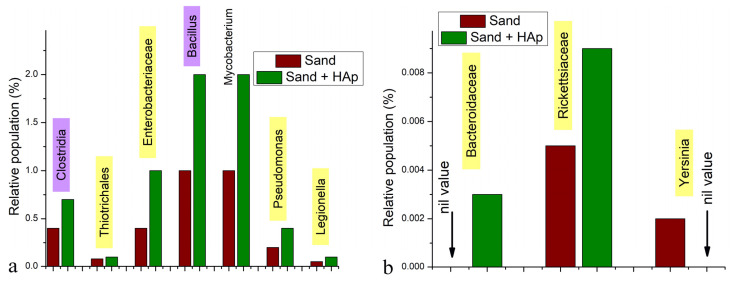
Relative presence of selected pathogenic bacterial taxa in the sandy soil microbiome present at concentrations higher (**a**) or lower (**b**) than 0.01% of the total microbiome before and after its treatment with HAp nanoparticles. Gram-negative taxa are framed in yellow, while Gram-positive taxa are framed in violet.

Regarding some of the common human pathogens present in the soil, the effect of HAp on most of them did not comply with the global trend of affinity for Gram-negative bacteria and the consequent boosting of the relative presence of their Gram-positive counterparts. For example, the Gram-negative family of Enterobacteriaceae, a member of the Gammaproteobacteria class, which includes the genera of *Escherichia*, *Salmonella* and *Shigella* (none of which were detected in the soil), increased in prominence, from 0.4 to 1% with the HAp treatment (Figure 8a). The most prevalent species in this family was *Enterobacter hormaechei*, which increased from 0.03 to 0.1% of the total microbiome with the HAp treatment. The order within the Gammaproteobacteria class to which the Enterobacteriaceae family belongs, namely Enterobacterales, includes *Yersinia pestis*, which causes the bubonic and pneumonic plague and is considered as the human pathogen in the West Coast soil with a highest potential to cause a pandemic in the future [56]. Although this species was not detected in the soil, just like its next of kin, *Y. enterocolitica*, its genus, *Yersinia*, was present at 0.002% of the total microbiome, but it became fully removed by the treatment with HAp (Figure 8b). Bacteria from the *Yersinia* genus are known for being facultative intracellular parasites causing a wide array of diseases, ranging from plague to Crohn’s disease to arthritis, and they have most likely been deposited by rodents and their fleas, which are common in the area [57]. Next, none of the species from the Gram-negative *Burkholderia* genus that are infective to humans and other mammals, such as *B. mallei*, *B. pseudomallei* or *B. cepacia*, were detected in the soil, and the most prevalent species were *B. glathei* (0.02%) and *B. bryophila* (0.01%), which both increased in prevalence with the HAp treatment, to 0.06 and 0.03%, respectively, just like the *Burkholderia* genus per se, which increased from 0.2 to 0.5%. Similarly, the Thiotrichales order of the Gammaproteobacterium class increased from 0.08 to 0.1% with the HAp treatment (Figure 8a), but the only distinctly detected species from it was *Candidatus marithrix* (0.007 and 0.009% for the untreated and treated microbiomes, respectively), and more pathogenic species, such as *Francisella tularensis*, the causative agent of rabbit fever, or *Francisella novicida*, the frequent cause of sepsis [58], were not detected.

The *Mycobacterium* genus also increased in prevalence with the HAp treatment (Figure 8a), but no common human pathogens belonging to it, such as any members of the *Mycobacterium tuberculosis* complex or *Mycobacterium leprae*, causing tuberculosis and leprosy, respectively, were detected. Collectively, however, all of the mycobacteria other than those causing tuberculosis and leprosy are known as nontuberculous mycobacteria, and they do cause pulmonary diseases resembling tuberculosis [59]. The most prevalent species in the microbiome were *M. confluentis* and *M. ilatzerense*, both at 0.04% in the untreated soil and at 0.06 and 0.05%, respectively, in the HAp-treated one. Moreover, the diversity of this genus doubled with the treatment, from five distinctly detected species in the untreated microbiome to 10 in the treated one, with species such as *M. madagascariense* (0.006%), *M. shinjukuense* (0.004%), *M. nonchromogenicum* (0.004%) and *M. obuense* (0.002%) counting among the newly detected, most likely due to the dispersion effects, which quadrupled the sensitivity, reducing the detection level from 0.008% for the least prevalent *Mycobacterium* in the untreated soil (i.e., *M. vaccae*) to 0.002% in the treated one. Next, the Gram-negative *Bacteroides* genus, the species of which make up the greatest portion of the mammalian gastrointestinal microbiota [60], but can also turn into opportunistic and antibiotic-resistant pathogens (e.g., *Bacteroides fragilis*) [61], was absent from the soil microbiome, but its family, Bacteroidaceae, became detectable after the treatment with HAp, at 0.003% of the total microbiome (Figure 8b), along with the families of Marinilabillaceae (0.004%) and Prevotellaceae (0.009%). Furthermore, the Epsilonproteobacteria class, which harnesses a pathogen such as *Campylobacter*, a cause of colitis in immunocompromised patients [62], was absent from the microbiome. Neither were *Actinomyces* detected in the soil nor any Spirochaete bacteria, such as those from the *Leptospira* genus or the Lyme disease-causing *Borrelia* genus. In addition, the only two genera within the Coxiellaceae family detected were *Ricketsiella* (0.1 and 0.4% for the untreated and the treated soils, respectively) and *Aquicella* (0.01% for both), and no pathogens from the *Coxiella* genus, such as *C. burnetii*, were detected. *Legionella*, a genus from the Gammaproteobacteria class and the cause of Legionnaires’ disease, was present in the soil microbiome at the concentration of 0.05%, which doubled to 0.1% with the HAp treatment (Figure 8a). No genera within the Rickettsiaceae family were discriminated, but the presence of this family harboring a number of pathogenic *Rickettsia* species causative of typhus, boutonneuse fever, rickettsialpox, Flinders Island spotted fever and other infectious diseases increased from 0.005 to 0.009% in the soil after the application of HAp (Figure 8b).

**Figure 9:**
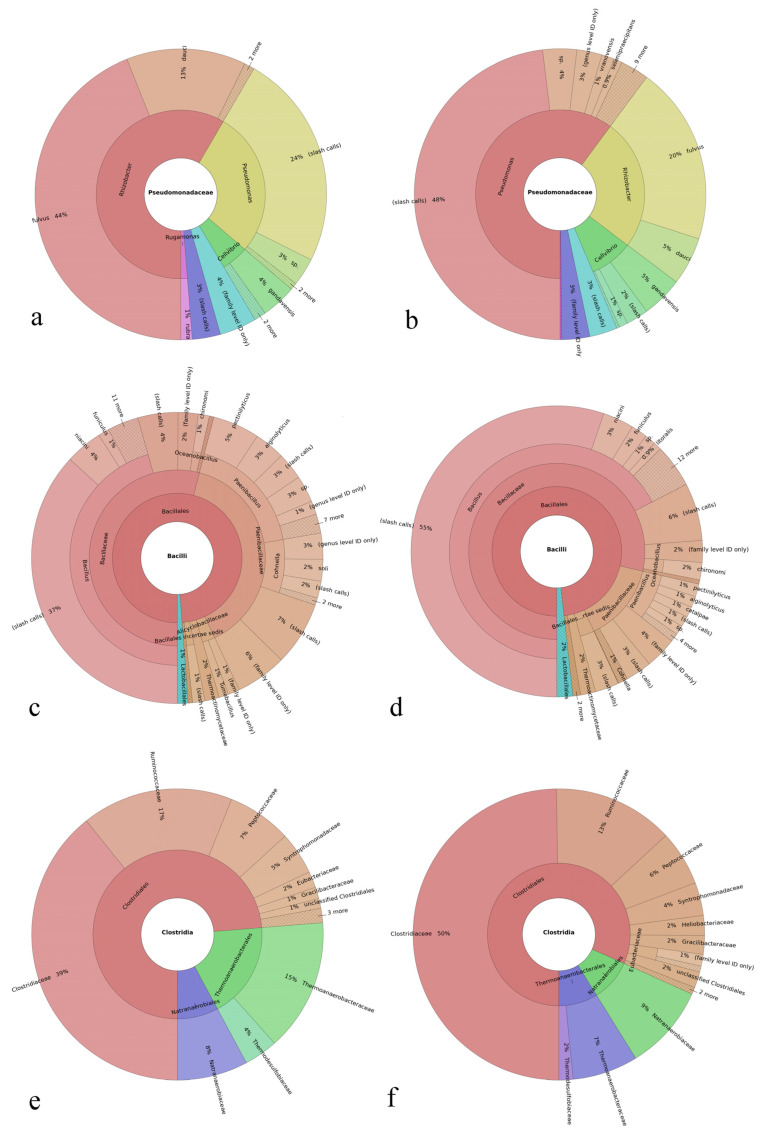
Krona plots focused on the bacterial groups containing some common human pathogens, including Pseudomonadaceae family (**a**,**b**), Bacilli class (**c**,**d**) and Clostridia class (**e**,**f**) for the untreated (**a**,**c**,**e**) and treated (**b**,**d**,**f**) soil samples.

Interestingly, *Pseudomonas*, another genus from the Gammaproteobacteria class, increased in prominence, from 0.2 to 0.4% with the treatment (Figure 8a) and while *P. vradovensis* was the only species precisely discriminated in the untreated soil, a number of other species were detected after the treatment, including the following, in the order of their prominence: *P. vranovensis*, *P. seleniipraecipitans*, *P. luteola*, *P. resinovorans*, *P. abletaniphilia*, *P. stutzeri*, *P. fuscovaginae*, *P. putida*, *P. rhizosphaerae*. *P. viridiflava*, and *P. xanthomarina*. Neither *P. aeruginosa* nor any of the species from the *P. aeruginosa* group except *P. resinovorans* [63] were detected. *Pseudomonas* species in general are soil bacteria adaptable to various environments, and the absence of both *P. aeruginosa*, the most common disease-causing Pseudomonas bacterium [64] (Figure 9a,b), and any staphylococci, such as *Staphylococcus epidermis* or *Staphylococcus aureus*, the opportunistic pathogens residing on human skin [65] (Figure 9c,d), excludes the possibility of contamination, suggesting the promoted dispersion and isolation effects achieved by HAp as the most probable reason for this counterintuitive amplicon enhancement. Namely, while Gram-negative species adhere onto the surface of HAp nanoparticles and as such become removed from the solution via centrifugation (if not being partially protected from lysis by mere adsorption onto nanoparticles that remain dispersed), there are competitive adsorption effects going on incessantly during the co-incubation period. These competitive dynamic effects may be largely analogous to the Vroman effect that protein adsorption is subject to [66]. In these scenarios, lighter and more motile bacteria would become initially adsorbed onto the particles but only to be replaced in subsequent stages by the bulkier and less diffusive bacteria, sending the former bacteria back into the medium, where they would be adopting a more dispersed form than initially, being as such more susceptible to detection. If the *Pseudomonas* species present such initially adsorbed and subsequently displaced bacteria, this may explain their elevated amplicons after the treatment with HAp nanoparticles in spite of the preference of these nanoparticles for Gram-negative microorganisms, to which *Pseudomonas* species belong. This may explain why the Pseudomonodaceae family becomes moderately diversified with the HAp treatment in contrast to the Bacilli class of pathogens, which reduces in diversity.

Next, both the relative population of the Bacillales order, which belongs to the Bacilli class, and the relative population of the *Bacillus* genus (Figure 8a) increased with the treatment with HAp, from 2 to 3% and from 1 to 2%, respectively, complying with the overall trend applying to Gram-positive bacteria. The most pathogenic of all *Bacillus* species existent in the West Coast soil is the anthrax-causing *Bacillus anthracis* [67], which was not detected either before or after the treatment among the 13 to 15 different *Bacillus* species detected, respectively, including *B. niacini*, *B. funiculus*, *B. kribbensis*, and *B. litoralis* as the most prevalent. Likewise, opportunistic pathogens from the genera of *Staphylococcus* or *Listeria* were not detected in the soil, neither before nor after the treatment.

Another class of pathogens monitored closely for potential removal with HAp was Clostridia (Figure 9e,f), which is generally ubiquitous in the soil [68] and which indeed was a part of the Bacilli class all until 1924 [69], when it became divorced from it, receiving its own class under the Firmicutes phylum, to which Bacilli belongs as well. Clostridia is a class of several known nosocomially acquirable pathogens, including those causative of gas gangrene (*C. perfringens)*, colitis (*C. difficile*, *C. perfringens*), tetanus (*C. tetani*), and botulism (*C. botulinum*, *C. butyricum* and *C. baratii*). It is widely accepted to be a Gram-positive bacterium, but because of its unique characteristics, it can also behave as Gram-negative. These unique characteristics are caused largely by the existence of the paracrystalline surface protein array, i.e., the S-layer, which serves a variety of functions and constitutes about 15% of the total protein makeup of a cell such as that of *C. difficile* [70]. Because of its rapid dissipation and replenishment, it was estimated to require the translation of around 500 molecules per second [71]. The outermost surface of the *C. difficile* cell is dominated by the low molecular weight (M_w_) S-layer protein, which plays a primary role in cell adhesion and is more variable than its innermost high M_w_ analogue, having no significant similarity to any other proteins [72]. The protein domain exposed on the surface contains loops with a high level of sequence variability and immune system evasion [73]. The relative population of Clostridia in the soil, however, increased from 0.4 to 0.7% after the treatment with HAp (Figure 6b and Figure 8a). The lack of attraction of HAp onto the entropic regions of proteins is instructive and may be a link to the increased antibacterial activity of HAp with respect to clinical, MDR strains of Gram-negative species than for their laboratory analogues [20] (Table 1). All in all, although HAp could remove the prominent Gram-negative bacterial taxa from the soil efficiently, it had the opposite effect on the less pervasive taxa, which happen to be those causative of disease in humans. One exception to this inversion of the trend was the effect on *Yersinia*, which was completely eliminated from the soil after its treatment with HAp (Figure 8b).

Here, it should be repeated that the observed increase in the populations of pathogens listed here with the treatment is an intrinsic methodological artifact, given that HAp, itself, cannot bring any more bacteria than there are in the system. Instead, as more prominent members of a taxon are abundantly removed from the system, the relative presence of the less abundant ones will increase as a consequence of this, even when they are being spared any interaction with HAp. Therefore, these deviations from the trend should not be taken for their face value, as all that they suggest is that HAp does not prefer to bind bacteria that are common human pathogens in the soil as compared to other bacteria in it.

### 3.5. Analysis of the Effect on Positive Bacteria in the Soil

A healthy microbiome is, before anything else, a diverse microbiome, and the ability of HAp to increase the diversity of all taxa tested (Figure 5), even if this is may be a mere technical effect, is a promising sign that HAp positively affects the microbiome. This deduction would be in line with a number of research studies testing HAp, often with success [74], as a form of phosphate fertilizer [75]. Still, the sparse solubility and alkaline nature of HAp have made other calcium orthophosphates, primarily the most acidic and soluble of them, calcium monophosphates [76], the fertilizers of choice among this family of compounds.

**Figure 10:**
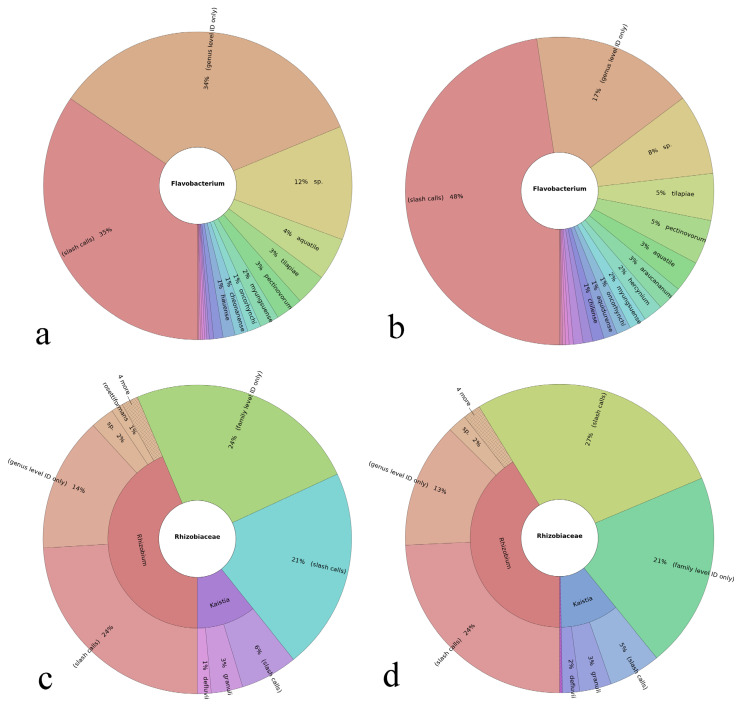
Krona plots focused on the *Flavobacterium* genus (**a**,**b**) and the Rhizobiaceae family (**c**,**d**) correlating positively with plant growth for the untreated (**a**,**c**) and treated (**b**,**d**) soil samples.

**Figure 11:**
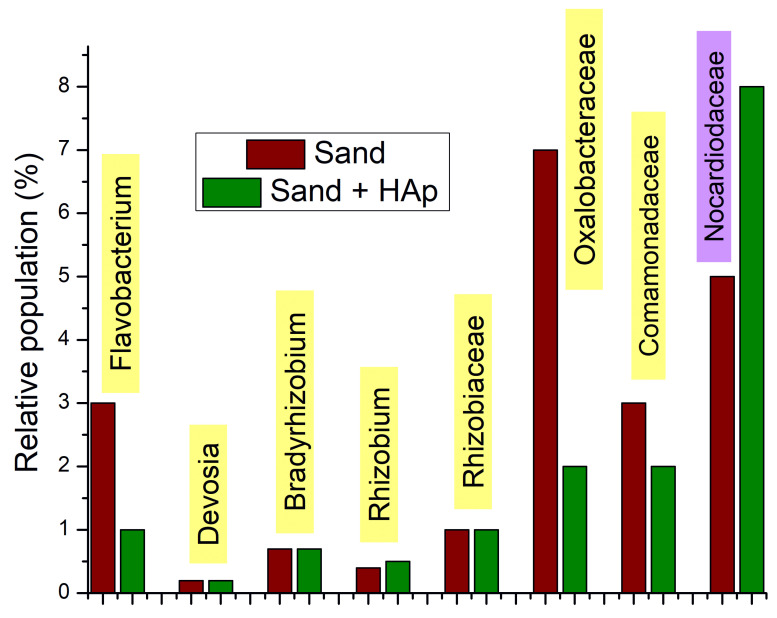
Relative presence of selected taxa positively correlating with the agricultural fertility of the soil before and after its treatment with HAp nanoparticles. Gram-negative taxa are framed in yellow, while the single Gram-positive taxon is framed in violet.

In addition to this global effect on the soil microbiome, there have been a number of bacterial taxa positively correlating with the plant growth promotion and/or transition from wild to domesticated soils. The effect of HAp on such taxa is summed in Figure 10 and Figure 11. *Flavobacterium* genus, for example, is a plant growth promoter and phytopathogen antagonist [77], but HAp decreased the population of *Flavobacterium* from 3 to 1% of the total microbiome, although it did mildly increase the total number of discriminable species, from 15 to 17. *Rhizobium* and *Devosia* genera also correlated positively with plant growth [78], and HAp increased the relative presence of Rhizobium from 0.4 to 0.5% and reduced the number of distinct species from five to four, while it did not affect the *Devosia* genus (0.2%) nor the Rhizobiaceae family (1%) to which a number of positive root bacteria belong. *Bradyrhizobium*, a common root-nodulating bacterium [79], was also unchanged in proportion (0.7%). Oxalobacteraceae and Comamonadaceae are two families of the Burkholderiales order containing positive plant bacteria [80], but their populations declined from 7 to 2% and 3 to 2%, respectively, with the HAp treatment. HAp, however, increased the relative population of the Nocardioidaceae family, from 5 to 8%, which is another family whose presence has been shown to increase with the transition from wild to domesticated soil. At the same time, HAp decreased the population of Cytophagaceae from 2 to 0.6%, which is a positive effect considering that this Bacteroidetes family correlates negatively with the domestication of the soil [81], as it has been associated with the overexpression of β-glucosidase, which is an enzyme involved in the degradation of cellulose [82]. Considering all of the taxa mentioned in this subsection, HAp reduced the population of each on average by 10.8 ± 5.4%.

The results presented in this and the previous subsection combined show that even though HAp has limited, if any, potential to diminish the pathogenicity of the soil from the human point of view, it is capable of promoting specific populations of bacteria responsible for the healthy crop growth and downregulating others that may have a detrimental agricultural effect. To a biological material, however, this ability to evade the pathogenic bacteria and bind more selectively to healthier ones in the microbiome might have proven itself as a crucial evolutionary advantage over other potential candidates for the inorganic component of hard tissues in mammals. Given that HAp of teeth is in most vertebrates one of the first structures that come into contact with the external sources of energy, having a material at the opening of the digestive tract that exhibits this type of selectivity may minimize the chances for pathogenic biofilm formation and foster a healthier microbiome in the mouth, the gut and the intestines.

### 3.6. Mechanism of Gram-Negative vs. Gram-Positive Selectivity

A logical question to be tackled at this point in the discussion is why HAp shows one intensely selective affinity for Gram-negative microorganisms as that evidenced here. One point that should be highlighted here is that this affinity must stem from the surface attraction. In other words, the chemical interaction between the surface of the bacterial cells and the surface of the nanoparticles must be subjected to scrutiny in order to explain the observed selectivity.

**Figure 12:**
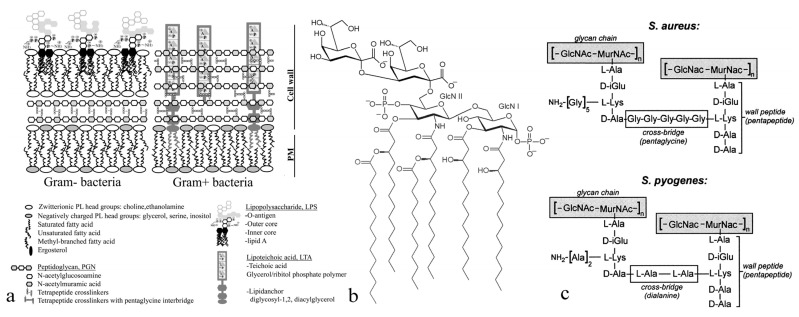
Schematic representation of the cell envelopes of Gram-negative and Gram-positive bacteria (**a**), along with the chemical structures of lipopolysaccharides (LPS, (**b**)) and peptidoglycans (**c**) forming them. The chemical structure of peptidoglycans is shown for two Gram-positive bacteria of choice: *Staphylococcus aureus* and *Streptococcus pyogenes*. Subfigures (**a**–**c**) are adapted and modified with permission from Refs. [96,97,98], respectively.

Indeed, one key structural difference between Gram-negative and Gram-positive bacteria is the existence of the lipopolysaccharide (LPS) bilayer membrane surrounding the cell wall composed of a thin peptidoglycan layer in the former and the absence of this membrane in the latter [83]. To compensate for the absence of this protective membrane, the cells walls of Gram-positive microorganisms comprise thicker peptidoglycan layers: 20–80 nm vs. 5–10 nm. The chemical difference between peptidoglycans and LPS is substantial: while both types of molecules integrate sugar moieties, the former are composed of amino acids, while the latter are amphiphilic lipids. Here, invoking sheer electrostatic effects to explain the difference in affinity would be too simplistic, even though these effects do normally provide the first barrier prior to achieving an optimal binding between colloidal entities [84,85]. HAp is mildly negatively charged under the physiological conditions [86], and it is known that positively charged nanoparticles are far better attractors of bacteria than their negative counterparts [87]. Gram-positive bacteria may be more negatively charged, as noted previously [88], and the low negative zeta potential and alternation of high-valence ions on the surface, i.e., Ca^2+^, HPO_4_^2−^ and PO_4_^3−^, may predispose HAp for a stronger attraction to the less charged Gram-negative surface. However, more direct molecular specificities are bound to be decisive in determining the outcome of this interaction. For this specific interaction to be elucidated, familiarity with the structure and orientation of LPS molecules forming the outer sheath of Gram-negative bacteria and of peptidoglycans forming the cell wall of Gram-positive bacteria is needed (Figure 12a).

As for the structure of LPS molecules forming the outer leaflet of Gram-negative bacteria, it consists of three distinct regions [89]: (1) lipid A on the innermost side, i.e., endotoxin, a glycolipid with the hydrophobic tail with the length of ≈10 nm, typically a β-1′,6-linked disaccharide of 2-amino-2-deoxy-D-glucose (D-glucosamine, D-GlcN) to which fatty acids, e.g., 3-hydroxyalkanoic acids, are attached via ester or amide linkages; (2) core oligosaccharide in the middle, commonly containing sugars such as 3-deoxy-D-*manno*-oct-2-ulosonic acid (K_do_) and L-*glycero*-D-*manno*-heptose (L,D-Hep); and (3) O-antigen, a distal polysaccharide composed of a number of distinct repetitive oligosaccharide (O) units on the outermost side (Figure 12b). Whereas the core oligosaccharides are crucial for mediating adhesion and cohesion, the O-antigen component is critical for imparting the proper elasticity to the bacterial cell [90], and the role of lipid A is to form activated complexes with the host cell [91]. While the O-antigen is the most variable part of the LPS molecule and is often used in strain sensing, the lipid A composition is greatly preserved among all Gram-negative bacteria, leading to wide serological cross-reactions [92]. In contrast, peptidoglycans of Gram-positive bacteria form a sacculus surrounding the cytoplasm as a single macromolecule consisting of glycan chains of alternating N-acetylmuramic acid and N-acetylglucosamine residues connected by short peptides composed of L-alanine, L-lysine, D-isoglutamine and pentaglycine attached to the epsilon amino group and having the terminal D-alanine-D-alanine group (Figure 12c). Furthermore, these stem peptides are cross-linked through the pentaglycine bridges with bonds that are reversible and tunable to the bacterial state, typically leading to reduced cross-linking during an infective state and denser cross-linking during the stationary phase [93] so as to provide the surface proteins with a greater mobility during the active stages of the bacterial lifecycle. In simplistic terms, therefore, the dynamics of the Gram-positive bacterial surface is predominantly a polypeptide phenomenon, whereas that on the surface of Gram-negative microorganisms is sugar-based. From the thermodynamic standpoint, peptidoglycans, because of their more pronounced peptide structure, should have a higher hydration enthalpy than sugars, which might predispose Gram-negative bacteria for a lesser potential for dispersion in the planktonic form. This presumably greater propensity of the Gram-positive surface for dispersion in the planktonic form is also augmented by its amphiphilic lipoteichoic acid moieties protruding the peptidoglycan layer (Figure 12a).

The plausibility of this argument that the lipoteichoic surface extensions play an important role in endowing Gram-positive bacteria with the ability to evade the capture by the nanoparticles may be supported by the inability of HAp to sequester mycobacteria (Figure 6e and Figure 8a), which fall outside of the classical Gram-positive vs. Gram-negative classification but contain similarly amphiphilic mycolic acid protrusions from the cell wall. Mycolic acid moieties are long-chain fatty acids protruding outwardly from the mycolyl–arabinogalactan–peptidoglycan complex that forms the envelope around the mycobacterial cell [94,95]. Similar to lipoteichoic extensions, they impart moderate surfactant properties to the bacterial cells, increasing their dispersibility. The greater tendency for dispersion, of course, translates to a greater probability for evasion of capture by the foreign solid phase, which here takes the form of HAp nanoparticles. At the same time, when the O-antigen of Gram-negative bacteria binds to HAp, its relatively rigidly hydrogen-bonded water molecules get released into the solution, which provides for a greater entropic gain than that achieved by the release of hydrogen-bonded water molecules from the looser hydration shell of a hydrophilic peptide upon its binding to HAp. This provides for yet another thermodynamic advantage in favor of binding HAp to Gram-negative microorganisms as compared to its binding to their Gram-positive counterparts.

Alongside electrostatics and thermodynamics, more direct chemical effects are bound to have a decisive say on the affinity of nanoparticles for the bacterial cell membrane, especially beyond the first few atomic layers of this membrane, should they be disrupted by the nanoparticles. Here, unlike the peptidoglycans of Gram-positive bacteria [99], the hydrophilic end of the canonic lipid A component of the membrane LPS molecules is phosphorylated, exposing phosphate groups along with carboxylic residues of the dodecanoic moieties [100], thus making the outer surface of Gram-negative microorganisms more structurally similar to the outer surface of the eukaryotic cells, to which HAp excellently binds. The attraction between phosphorylated glucosamine units and carboxyl groups of the LPS molecules to calcium ions of HAp as well as the potential attraction between protonated amide groups of LPS and phosphates and/or hydroxyls of HAp may be the molecular basis for the attraction of Gram-negative bacteria to HAp. Here, calcium-mediated interactions may be the most probable mediators of the binding of HAp to LPS molecules, if we only remember that calcium and magnesium cation displacement presents the key feature of the mechanism of action of LPS-binding polymyxin antibiotics targeting the outer membrane of Gram-negative bacteria [101]. Moreover, the cell wall of bacteria is not a static structure but rather a rapidly renewable one, where LPS and other molecules constantly degrade and are replaced by new ones. For example, the most distal, O-antigen component of the LPS molecules is transported to the transmembrane region together with an undecaprenyl pyrophosphate moiety before it detaches from it and ligates to the LPS core [102], which may serve as another attractor to calcium cations of the HAp surface and of the hydrodynamic sphere around it. Adding up to this dynamic, fluctuant and permeant structure of the cell envelope, Gram-negative bacteria possess porins and with the earlier evidenced ability of HAp to penetrate into the bacterial cells of *E. coli* and localize inside it [21,103], this more potent internalization may be an additional factor favoring the affinity observed here.

### 3.7. Mechanism of Selectivity within the Gram-Negative Niche

Additionally, even within the Gram-negative niche, HAp exhibits notable selectivity, affecting certain taxa more than the others. In trying to understand the mechanistic reasons underlying this selectivity, a closer look at the few bacteria completely removed or removed by a multifold margin may be instructive. These bacteria include members of the Flavobacteriia class, the Oxalobacteraceae family and the *Phenylobacterium* and *Adhaeribacter* genera. Expectedly, all of these bacteria are Gram-negative. Limited information existing on their structural characteristics, however, limits the search for key factors determining their attraction to HAp. Additionally, Gram-negative bacteria that have not been eliminated can also be instructive, assuming that they must harness a protective mechanism that other Gram-negative bacteria prone to removal do not possess. Some of the essential characteristics of Gram-negative bacteria with the affinity for HAp and Gram-negative bacteria inert to it are listed in Table 2 and Table 3, respectively. Legionellales was the order whose relative prevalence highly increased after the treatment with HAp (Figure 6c), but because it comprises exclusively intracellular bacteria, which require external hosts, ranging from amoeba to humans, to proliferate, it was left out of this analysis.

By comparing the information listed in Table 2 and Table 3, it can be seen that metabolic characteristics vary within each group of bacteria and no correlations could be established with the propensity for removal by HAp. All bacteria effectively removed from the microbiome were either filamentous or rod-shaped, but a number of bacteria unaffected by HAp were also rod-shaped. One characteristic here, however, stands out: namely, every rod-shaped bacterial taxon whose members were not affected by HAp is typified by flagella-driven motility, and the only non-motile bacterium unaffected by HAp was a coccus. In contrast, most bacteria sensitive to HAp were either non-motile or motile by gliding. Gliding, which bacteria from the Flavobacterii class engage in (Table 2), is a form of movement that is not as active as the movements supported by flagella, pili or fimbriae, relying on the proton motive force within coupled inner membrane motor proteins and transmembrane proteins attached to the surface [104]. This suggests that the combination of (i) elongated shape and (ii) passive motility favors the binding of bacterial cells onto HAp. As for (i), this is understandable from the geometric point of view [105] simply because of the greater area of contact between particles and cells when neither of them are spherical. When both the cells and the particles adopt elongated morphologies, the surface area of their contact will be higher on average than when at least one of them adopts the shape of a sphere. As for (ii), this evokes the aforementioned Vroman effect as the explanation why HAp is highly effective against *Pseudomonas* in monoculture [20,21,22], but it does not bind it under the microbial community conditions measured here. According to this effect, more mobile entities tend to reach a foreign surface first and adsorb on it, but only to be later displaced by the less mobile, if not also bulkier, entities, which have a greater thermodynamic affinity for binding. This is a universal principle that applies not only to macromolecules but also to smaller molecular groups [66].

**Figure 13:**
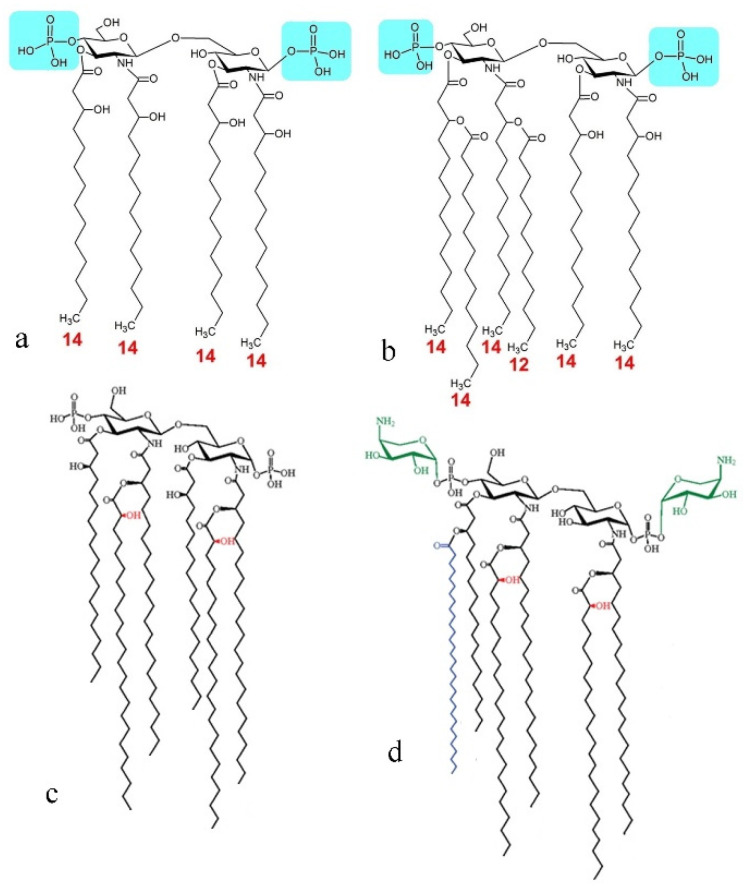
Lipid A portions of the LPS from *Yersinia pestis* (**a**), *Escherichia coli* (**b**), and two different forms of *Pseudomonas aeruginosa*: form A (**c**) and form D (**d**). Adapted with permission from Refs. [107,108].

Since a lot of metabolic assay data (Table 2 and Table 3) overlap between the bacteria affected by HAp and the bacteria unaffected by it, it is clear that the crucial characteristics must be sought at the morphological and surface structure levels. As for the surface structure, it is dominated by LPS, which forms the outer part of the Gram-negative bacterial cell envelope. Now, as for the most distal portion of the LPS, namely lipid A, it shows very little variability and is least likely to be a site of this selectivity. This is illustrated in Figure 13, showing the little different lipid A structures of a bacterium from the *Yersinia* genus (Figure 13a), which was removed by HAp, and a bacterium from the Enterobacteriaceae family (Figure 13b), which had no affinity for HAp, but also very different structures of lipid A in a single strain of *P. aeruginosa* depending on the environmental conditions (Figure 13c,d). The outermost, O-antigen portion of LPS, on the other hand, is subject to compositional heterogeneities that vary even between strains [106], let alone species. Although the additions of sugar moieties, such as glucosyl and fucosyl, and of groups such as acetyl and methyl count among the routine modifications that can alter the specificity of the interaction with a material such as HAp, the extreme variability of the O-antigen makes it an equally improbable source of this selectivity amongst Gram-negative bacteria. Still, there are certain structural characteristics of LPS that should be considered before variations to its sequence can be discarded with confidence as a factor of influence.

For example, one key aspect of the LPS coating of *Phenylobacterium* is the replacement of glucosamine, which is typical for Gram-negative bacteria, with 2,3-diamino-2,3-dideoxy-D-glucose in the lipid A backbone, as well as the presence of ester-linked 3-hydroxy-5-*cis*-dodecanoic acid, which makes this genera unique in the bacterial world [109]. As for Flavobacteriales, it is similar to Cytophagales, another order within the Bacteroidetes phylum, in that they both contain flexirubin pigment in the outer LPS sheath [110]. Flavobacteria also illustrate the ability of such additional ingredients of the cell wall to bind to the particle surface, given the demonstrated capacity of flavolipin of *F. meningosepticum* to compete with LPS molecules for macrophage receptors [111]. Therefore, one realistic possibility is that such additional membrane or transmembrane molecular entities could predispose some bacterial taxa to a more or less intense removal by HAp. This is especially so in view of the similarity in the chemical composition of carbohydrates forming the most variable, O-antigen portion of the LPS across the whole spectrum of Gram-negative bacteria. For example, one of the members of the Oxalobacteraceae family, *Naxibacter alkalitolerans* has been shown to have the O-antigen composed of the repeated units of 2-amino-2,6-dideoxy-galactose (fucosamine, FucN), 3-amino-3,6-dideoxy-glucose (quinovosamine, Qui3N), 6-deoxy-mannose (rhamnose, Rha) and galactose (Gal), with an acetyl group on FucN and a butirroyl group on Qui3N [112]. A member of the Flavobacterii class, *Flavobacterium psychrophilum*, has the O-antigen composed of a trisaccharide repeating units of L-rhamnose (L-Rha), 2-acetamido-2-deoxy-L-fucose (L-FucNAc) and 2-acetamido-4-R-2,4-dideoxy-D-quinovose (D-Qui2NAc4NR), where R represents a dihydroxyhexanamido derivative [113,114]. Another species from the same genus, *Flavobacterium columnare*, has the trisaccharide O-antigen composed of very different sugars, namely 2-acetamido-2-deoxy-dglucuronic acid (d-GlcNAcA), 2-acetamidino-2,6-dideoxy-l-galactose (l-FucNAm) and 2-acetamido-2,6-dideoxy-d-*xylo*-hexos-4-ulose (d-Sug) at the ratio of 1:1:1. Bacterial taxa showing no affinity for HAp, moreover, often share the compositional features with taxa exhibiting this affinity, with similar glycoforms, nonstoichiometric modifications and acetyl and phosphatidyl substitutions occurring in species such as *P. aeruginosa* [115]. As far as taxa inert to HAp are concerned (Table 3), *Bacteroides fragilis* from the Bacteroidaceae family has a short O-antigen saccharide chain composed of L-rhamnose, D-glucose, and D-galactose, with the antigenic specificity being determined by a β-1,6-linked D-galactose oligomer [116]. Next, the O-antigenic polysaccharide of *Rhizobium etli* was found to be relatively low-M_w_ glycan where each trisaccharide repeating unit consisted of glucuronic acid, fucose, and 3-*O*-methyl-6-deoxytalose [117]. The O-antigen of *Enterobacter cloacae* also consists of substituted L-Rha, DGLc, L-FucNAc, D-GlcNAc and D-Man units [118]. One characteristic surface feature of Enterobacteriaceae is an exopolysaccharide taking the form of a trisaccharide covalently linked to phosphoglyceride and is known as colanic acid, serving as an antigen additional to the O-antigen of the LPS.

All this is to say that although side chains of LPS do present the dominant surface antigen [119], it is uncertain if these chemical structure changes can be the decisive factor determining the presence or absence of affinity for HAp, especially since this interaction does not rely on the same molecular recognition specificities that reactions between molecular receptors and substrates rely on, but rather on the grosser physicochemical bonds. A more realistic scenario, therefore, is that specific surface structuring formed through the interaction of LPS with other molecules creates characteristic spacing between hydrogen bonding or electrostatic attraction centers that more or less fit the spacing between calcium cations, phosphate anions and hydroxyl groups on the terminal surface of HAp that comes into contact with the bacterium. The spatial complexity of this process requires similarly complex simulations, if not high-resolution imaging, before the selectivity experimentally discovered here can be understood on the atomistic level. The most realistic scenario, however, would imply an unselective interaction between HAp and Gram-negative bacterial cells, where, simply, cells with more developed modes of motility on average spend less time adsorbed onto HAp nanoparticles before they move on than cells that are either non-motile or are primitive gliders. Under such conditions, on average, after a finite span of time and at the point of separation of HAp from the system, an equilibrium is reached where the less motile species reside on HAp nanoparticles more copiously than their more sophisticatedly motile counterparts.

### 3.8. Potential Use for HAp as a Microbiome Balancer or Remediation Agent

The results presented here show that HAp substantially affects the soil microbiome used in these analyses as a model microbial community system. Although HAp has neither been able to selectively sequester human pathogens from the soil nor to leave the bacteria correlating positively with the crop growth fully intact, it did demonstrate a highly selective ability to bind and remove Gram-negative microorganisms from the soil without affecting their Gram-positive counterparts. Therefore, a most immediate use for HAp may be in rebalancing the microbial composition of soils infested with excessive amounts of Gram-negative microbes. Considering the reduction in the presence of Gram-negative species down to 60.8% with a single treatment (Figure 7b), which is equivalent to the factor of 1.65, it can be estimated that with ten successive treatments, the level of Gram-negative microorganisms in the experimental soil would be brought down to trace amounts (<1%).

Here, it is worth noting that soils contaminated by heavy metals, for example, do not only tend to undergo a drop in the diversity of their microbiomes, but they also display a more pronounced prevalence of Gram-negative Proteobacteria than the uncontaminated soils [120,121,122]. Similarly, bacterial communities in soils treated with glyphosate, an organophosphate herbicide widely used in agriculture, become dominated by Gram-negative bacteria [123] just as well as microbiomes of soils amended with animal waste fertilizers, such as chicken manure, do [124]. For such soils, the application of HAp as a microbiome balancer may be meaningful. In contrast, soils polluted by petroleum spills are characterized by an overabundance of Gram-positive species [125], most of which engage in the decomposition of oil [126], and HAp would not have a meaningful application niche there. Neither would it be applicable in the treatment of soils that exhibit an overabundance of Gram-positive bacteria due to the amendments with ammonium sulfate or certain types of sewage sludge [127]. Similarly, stress due to drought and rewetting has been shown to reduce the population of Gram-negative microorganisms and increase the Gram-positive populations [128], which are the very same effects as those achieved by HAp, meaning that its application to amend these changes to the microbiome would be redundant and ineffective.

Next, uses in water remediation, to clear up waters contaminated by bacteria, which are often predominantly Gram-negatives, are conceivable, too. For example, dental unit water supplies are most commonly contaminated by *Pseudomonas* and *Legionella* among other Gram-negative bacteria [129]. Aerator water supplies are also most commonly contaminated by *Legionella pneumophila* and other Gram-negatives [130], presenting the major source of infection for immunocompromised patients in hospitals. Even outside the clinical setting, Gram-negative species led by those from the *Pseudomonas* genus present the leading cause of contamination of public water supplies after various stages of the purification process [131]. In fact, the contamination of hot tubs, swimming pools and other public water recreation facilities is most often caused by Gram-negative microorganisms [132]. The same applies to physiological microbiomes, which may also be balanced with the use of HAp, especially in cases where Gram-negative opportunists have overpopulated the system and created a dysbiotic imbalance, as it is often seen in conditions such as Gram-negative bacteremia, pneumonia or urinary tract infections, which are in over 90% of cases caused by Gram-negative microorganisms [133]. The gut microbiomes of patients with cancer, diabetes mellitus or chronic kidney disease also often shift toward more Gram-negative when compared against the healthy individuals [134,135]. Knowing that virtually all of the bacteria beneficial for the gut health [136] are Gram-positive, the prevalence of which HAp elevates relative to that of their Gram-negative counterparts, HAp could indeed be proposed for the balancer of the gut microbiome were it not only for the relatively highly acidic conditions inside the stomach, under which HAp would almost momentarily dissolve. Additionally, it has been a long-standing paradigm in the field of dentistry that the progression of periodontitis is entailed by a shift from an oral microbiota consisting primarily of Gram-positive aerobes to the one comprising mostly Gram-negative anaerobes [137]. HAp, in the form in which it was tested here, is not able to delineate pathogenic from nonpathogenic species, but it is capable of altering microbiomes by exerting a gross and global attractive effect on large populations of Gram-negative microorganisms. Such a mode of action may often turn out to present the smoothest and the least side effect-inducing means of preventing or treating a disease that is caused in its starting stages by a quiescent shift in the microbiome composition.

## 4. Conclusions

Introduced into a complex bacterial microbiome network present in the sample of sandy soil obtained from a local playground, HAp substantially altered its composition. Around 220 distinct families, 290 genera and 580 species were discriminated and the number of operational taxonomic units increased by 7–13% upon the treatment with HAp, which was primarily due to augmented dispersion through competitive binding among other effects. Principal coordinate analysis demonstrated substantial changes to the microbiome composition to have occurred due to the treatment with HAp. Most importantly, HAp selectively bound Gram-negative bacteria from the soil microbiome. As a result, after separating HAp from the system, together with the bacteria bound to it, the concentration of Gram-negative microorganisms in the microbiome dropped and the relative amount of the Gram-positive bacteria increased. The greater affinity for the LPS of the Gram-negative cell envelope than for the peptidoglycans of the cell wall of Gram-positive bacteria was invoked in a model built on combined thermodynamic, electrostatic and chemical bond arguments. Microbial cells decorated with amphiphilic protrusions, such as lipoteichoic acids of Gram-positive bacteria or mycolic acids of mycobacteria, and the cells equipped with flagella have been shown to be more effective in resisting the capture by the solid phase than those devoid of these dispersive and motive structures. Common opportunistic taxa potentially infectious to humans were present at low concentrations in the microbiome, and they diverged from this dominant trend for the most part, given that HAp preferred binding to less pathogenic bacteria. Based on this, it can be supposed that HAp evolved as the biological material that provides a maximal microbiome balancing advantage for life forms whose sustainability is dependent on the health of the microbiomes harnessed internally and environmentally. Needless to add, more comprehensive research on various other microbiomes of interest other than the model one tested here are needed to further strengthen this idea. If proven correct in these other models, this would throw light on a new avenue for biomedical applications of HAp. Adding up to its traditional use for the promotion of biocompatibility, bioactivity and osteoconductivity of biomaterials, these findings pave the way for a potential new application for HAp in tissue engineering constructs, namely that of a microbiome balancing agent. It is likely that this new application for HAp will become more prominent as medical approaches to disease management begin to increasingly rely on microbiome manipulation as the way of treating and preventing disease. We are still far from that era, but the current interest in research on microbial communities takes us away from the ‘one bug, one disease’ model and nearer to these horizons each day.

## Figures and Tables

**Table 1:** IC_50_ values for HAp against regular, lab strains and multidrug-resistant (MDR) strains of different Gram-negative and Gram-positive bacteria, as determined from concentration-dependent viability curves in Luria/Vegatone broth assays [20].

Gram Stain	Species	Lab Strain	MDR Strain
		**IC_50_ (HAp) (mg/mL)**	**IC_50_ (HAp) (mg/mL)**
**Gram-negative**	*E. coli*	29	/
	*P. aeruginosa*	59	10
	*S. aureus*	>100	98
**Gram-positive**	*S. epidermis*	>100	/
	*E. faecalis*	93	/

**Table 2:** Selected Gram-negative bacterial taxa strongly affected by HAp.

Taxon	Motile	Shape	Catalase-Active	Oxidase-Active	Urea Hydrolysis	Esculin Hydrolysis	Citrate Utilization	Casein Hydrolysis
Flavobacterii	+(glide)	Filamentous	+	+	+	+	+	+
Oxalobacteraceae	+/−	Rods	+	+	+/−	+/−	+/−	+/−
*Phenylobacterium*	–	Rods	+	+	–	–	+	–
*Adhaeribacter*	–	Rods	+	+	+	+	–	+

**Table 3:** Selected Gram-negative bacterial taxa not affected by HAp.

Taxon	Motile	Shape	Catalase-Active	Oxidase-Active	Urea Hydrolysis	Esculin Hydrolysis	Citrate Utilization	Casein Hydrolysis
Bacteroidaceae	+(flagella)	Rods	–	+	–	–	+	+
*Enterobacter*	+(flagella)	Rods	+/−	–	+	+	+	+
*Rhizobium*	+(flagella)	Rods	+	+	–	+	–	+
*Amaricoccus macauensis*	–	Coccus	+	+	+	–	–	n.d.

## Data Availability

Data will be available upon reasonable request.
